# Supplementary figures and images for: Massively parallel analysis of human 3′ UTRs reveals that AU-rich element length and registration predict mRNA destabilization
Source: G3 (Bethesda). 2021 Nov 29;12(1):jkab404. doi: 10.1093/g3journal/jkab404 (PMC8728028; doi:10.1093/g3journal/jkab404)

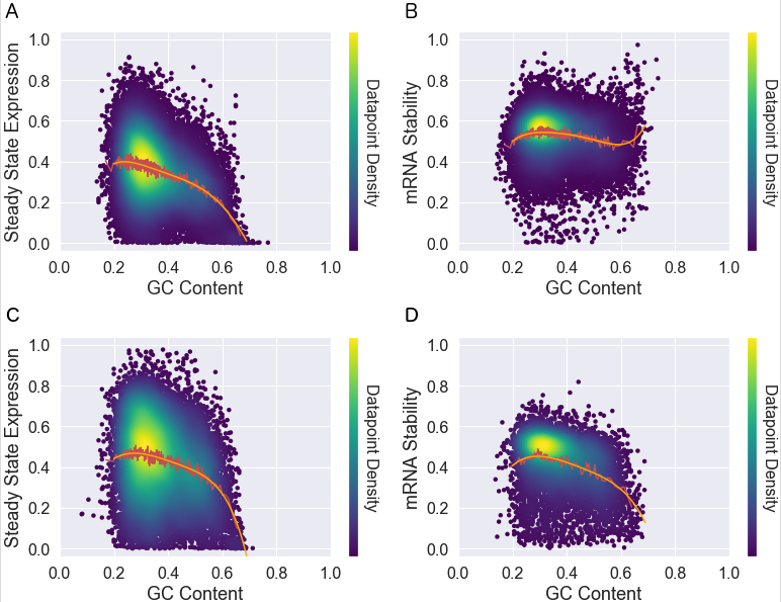

Supplement: jkab404_Supplementary_Figures [file jkab404_supplementary_figures.zip › GENETICS-G3-2021-402854-s04.tif]

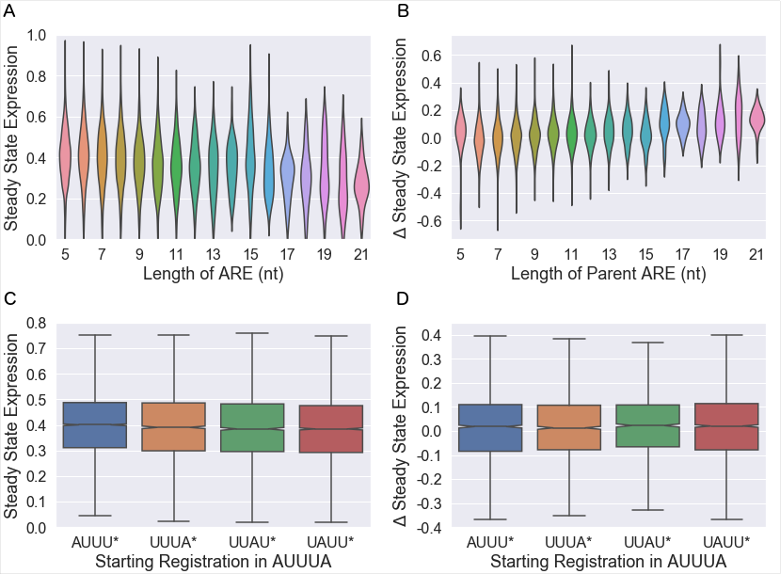

Supplement: jkab404_Supplementary_Figures [file jkab404_supplementary_figures.zip › GENETICS-G3-2021-402854-s05.tif]

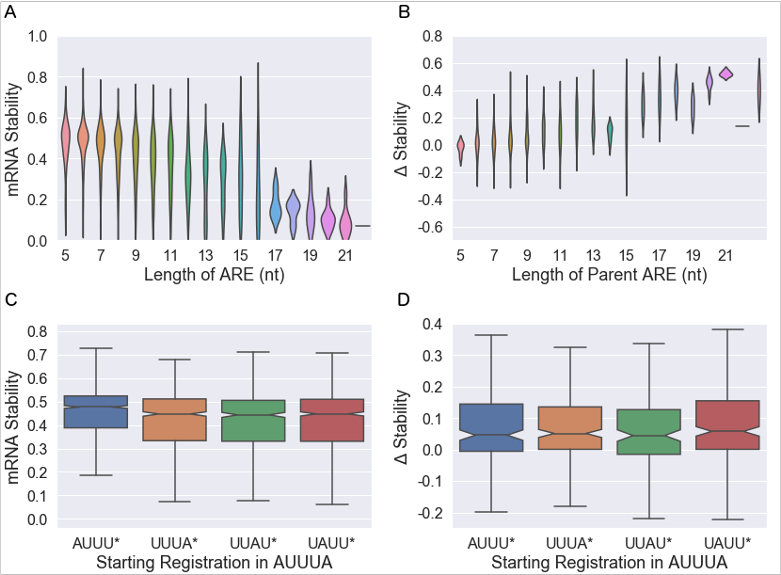

Supplement: jkab404_Supplementary_Figures [file jkab404_supplementary_figures.zip › GENETICS-G3-2021-402854-s06.tif]

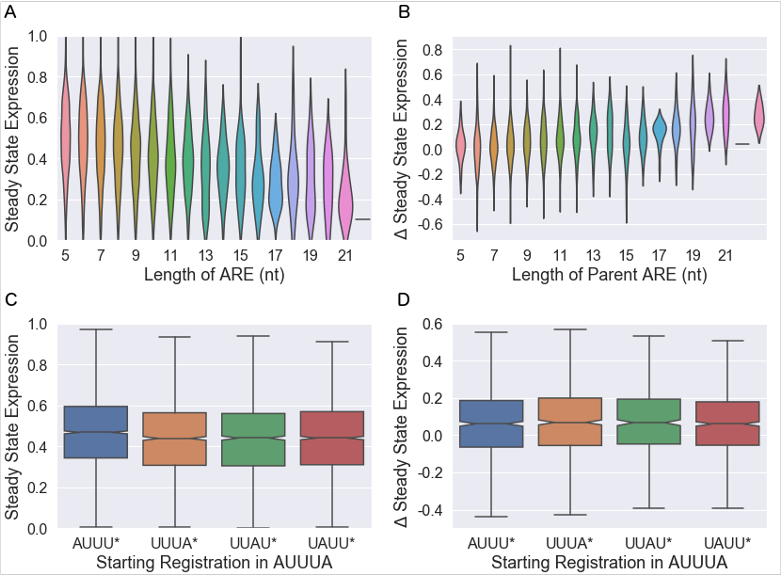

Supplement: jkab404_Supplementary_Figures [file jkab404_supplementary_figures.zip › GENETICS-G3-2021-402854-s07.tif]

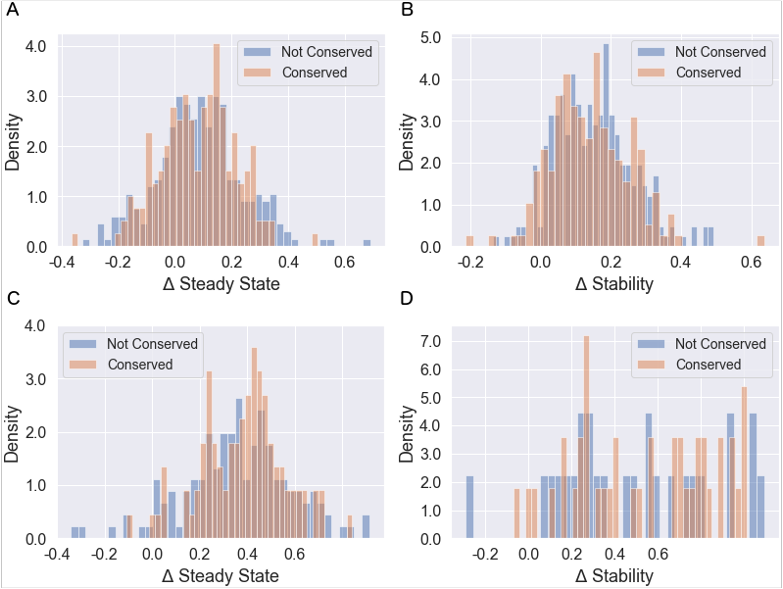

Supplement: jkab404_Supplementary_Figures [file jkab404_supplementary_figures.zip › GENETICS-G3-2021-402854-s08.tif]

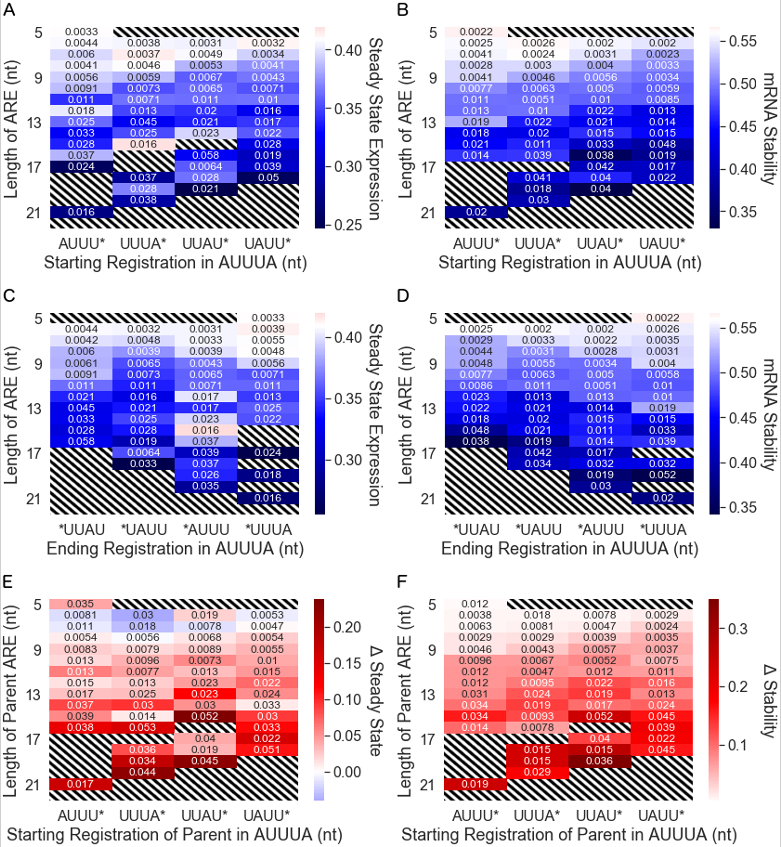

Supplement: jkab404_Supplementary_Figures [file jkab404_supplementary_figures.zip › GENETICS-G3-2021-402854-s09.tif]

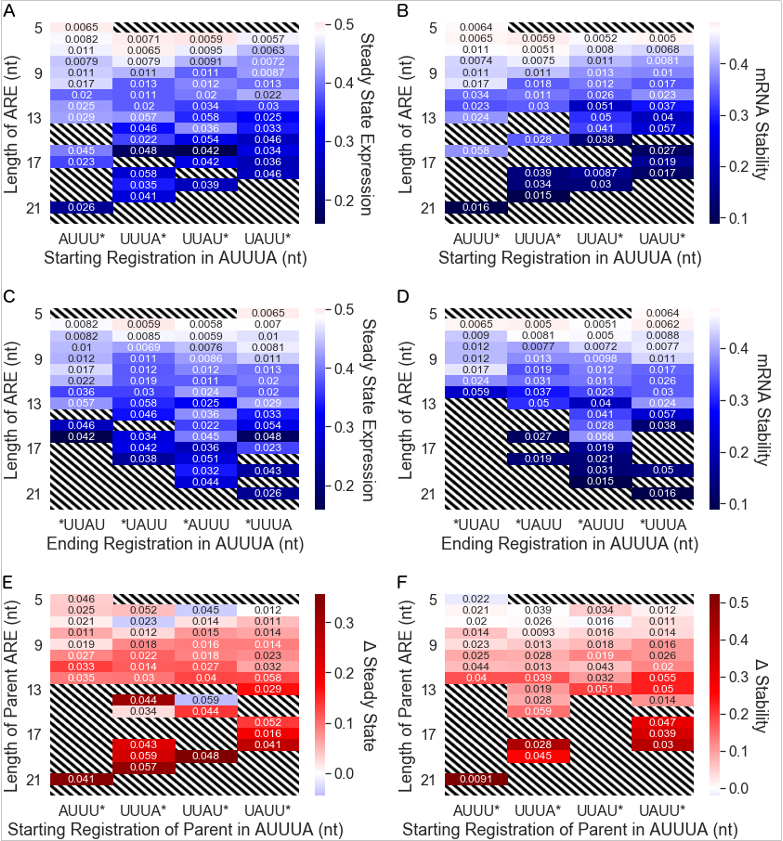

Supplement: jkab404_Supplementary_Figures [file jkab404_supplementary_figures.zip › GENETICS-G3-2021-402854-s10.tif]

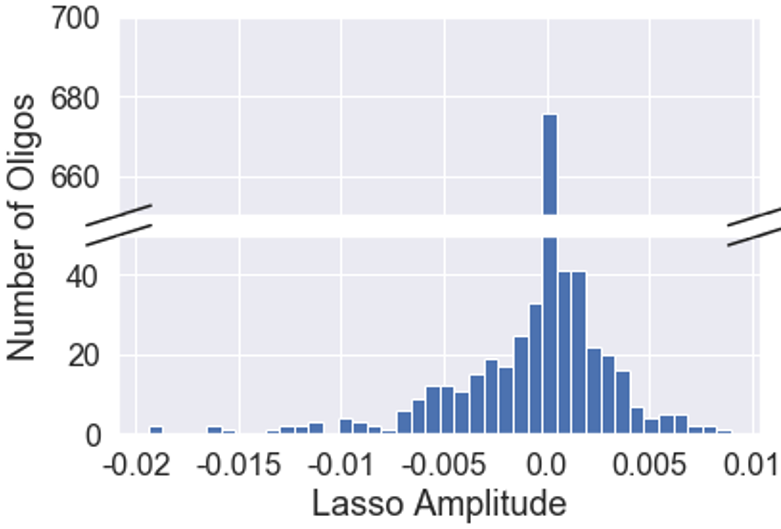

Supplement: jkab404_Supplementary_Figures [file jkab404_supplementary_figures.zip › GENETICS-G3-2021-402854-s11.tif]

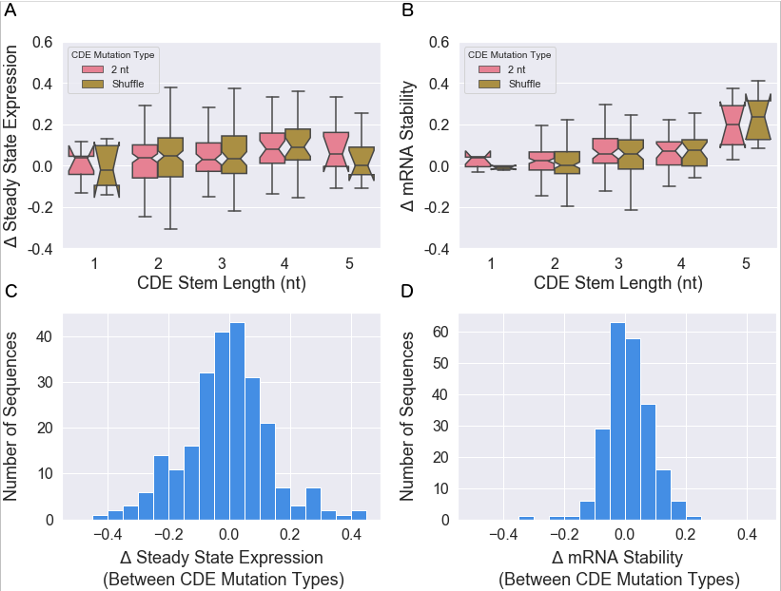

Supplement: jkab404_Supplementary_Figures [file jkab404_supplementary_figures.zip › GENETICS-G3-2021-402854-s12.tif]

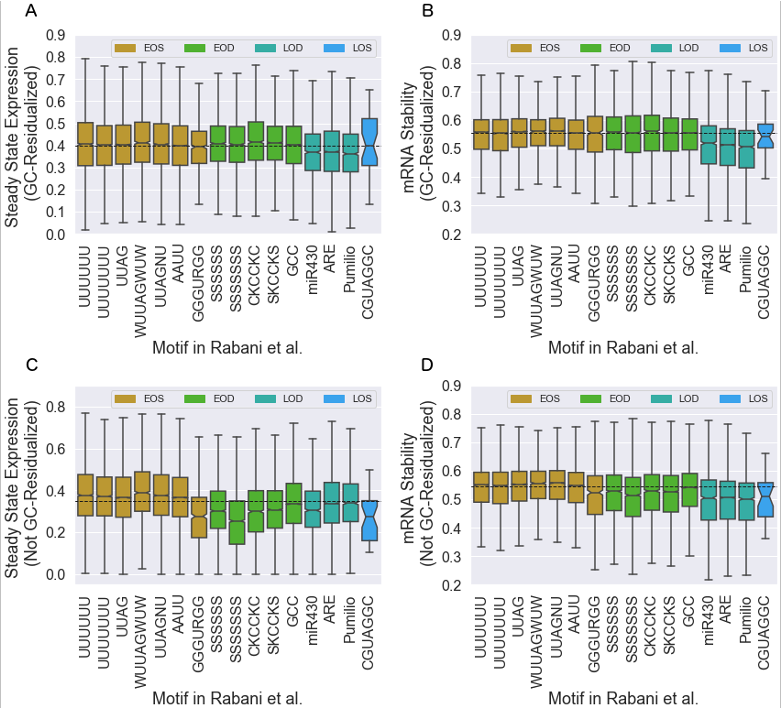

Supplement: jkab404_Supplementary_Figures [file jkab404_supplementary_figures.zip › GENETICS-G3-2021-402854-s13.tif]

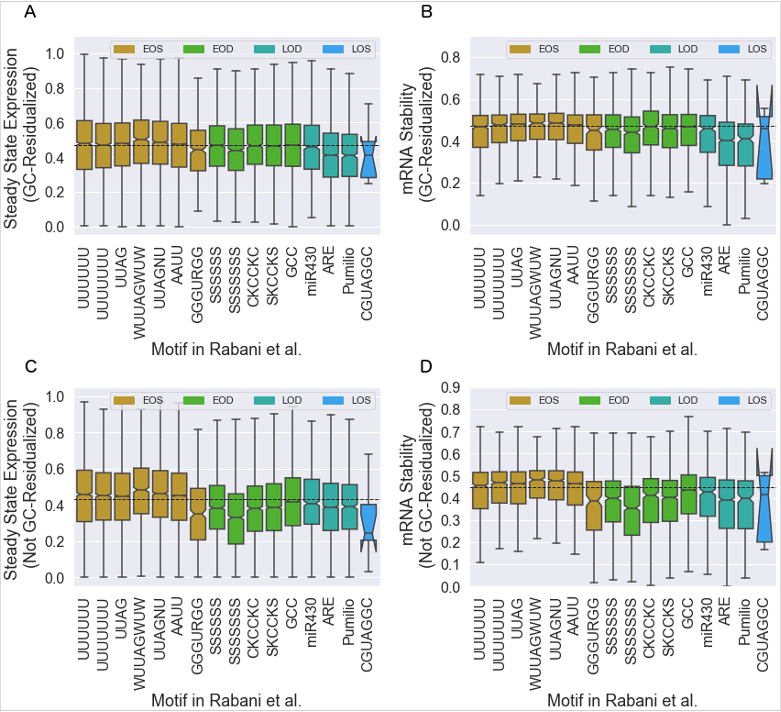

Supplement: jkab404_Supplementary_Figures [file jkab404_supplementary_figures.zip › GENETICS-G3-2021-402854-s14.tif]

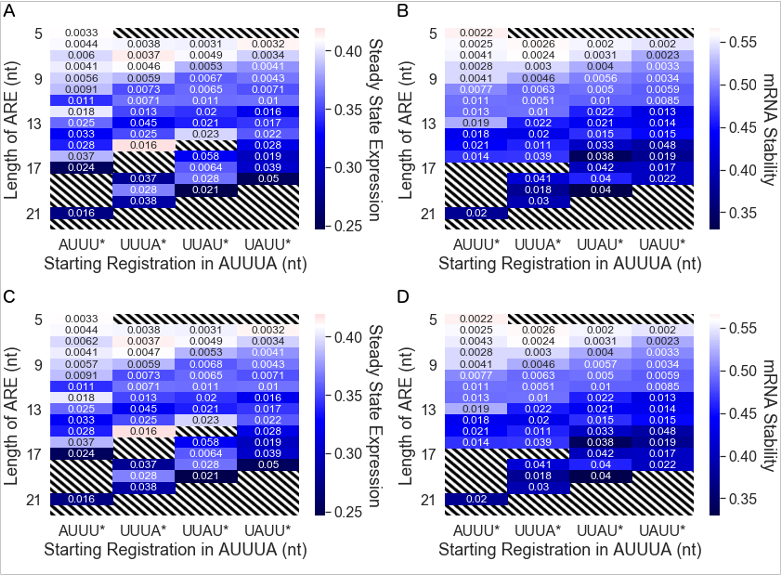

Supplement: jkab404_Supplementary_Figures [file jkab404_supplementary_figures.zip › GENETICS-G3-2021-402854-s15.tif]

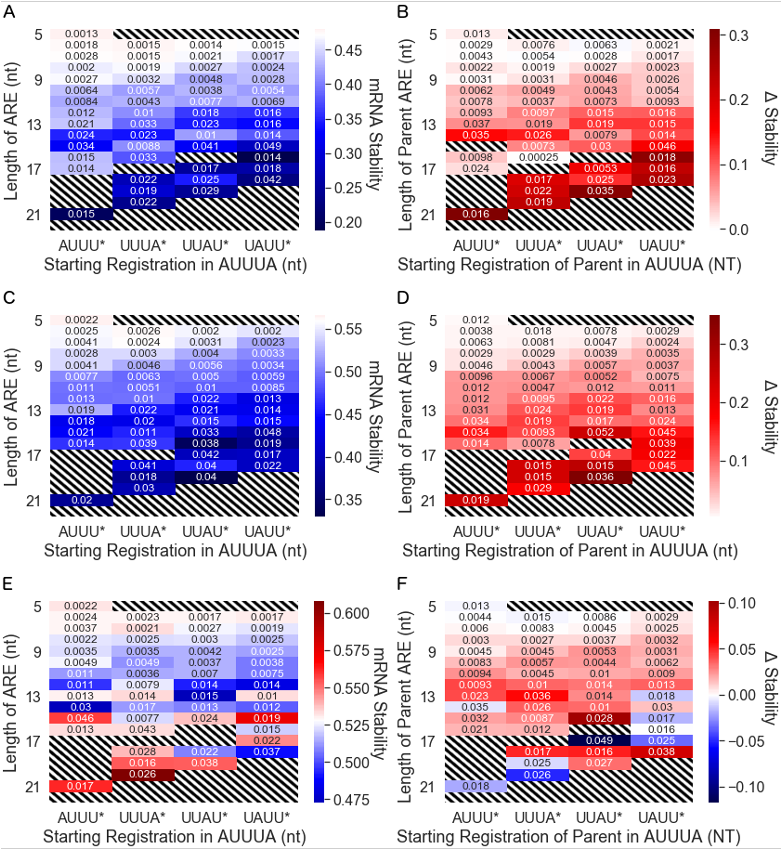

Supplement: jkab404_Supplementary_Figures [file jkab404_supplementary_figures.zip › GENETICS-G3-2021-402854-s16.tif]

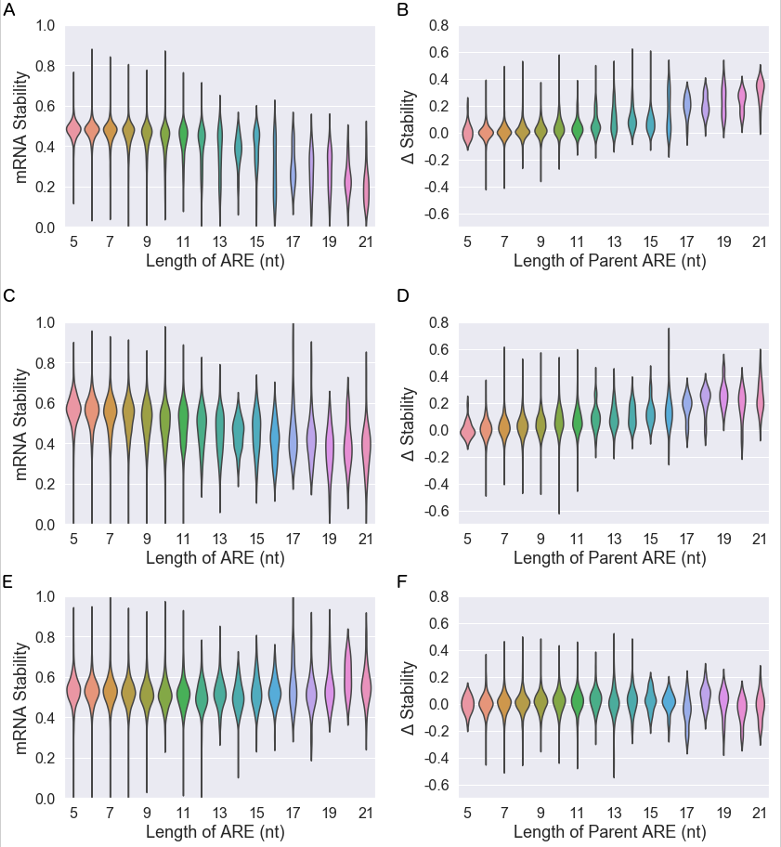

Supplement: jkab404_Supplementary_Figures [file jkab404_supplementary_figures.zip › GENETICS-G3-2021-402854-s17.tif]

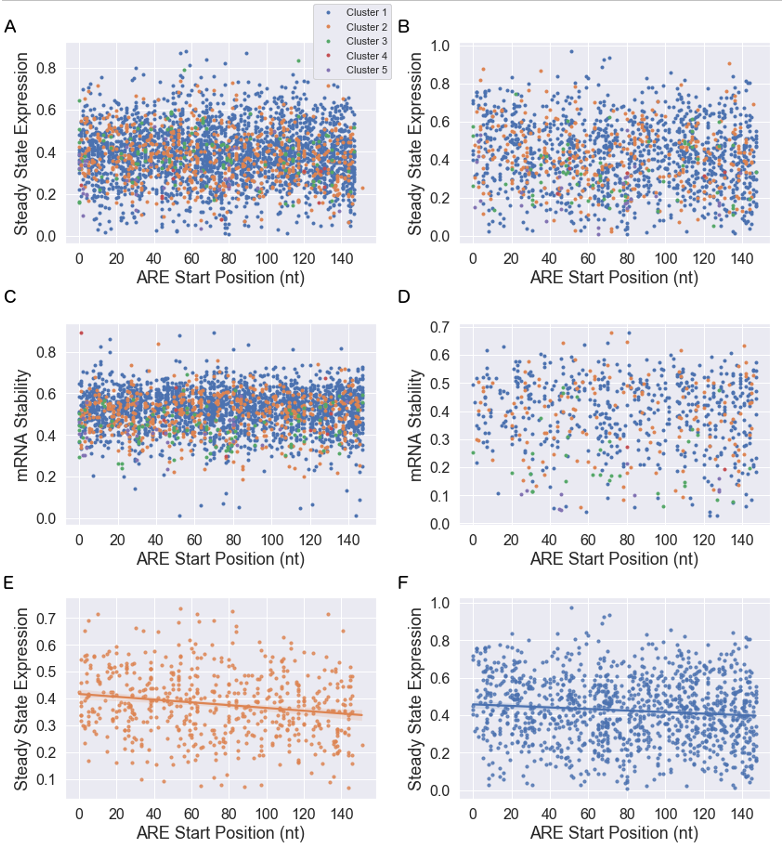

Supplement: jkab404_Supplementary_Figures [file jkab404_supplementary_figures.zip › GENETICS-G3-2021-402854-s18.tif]

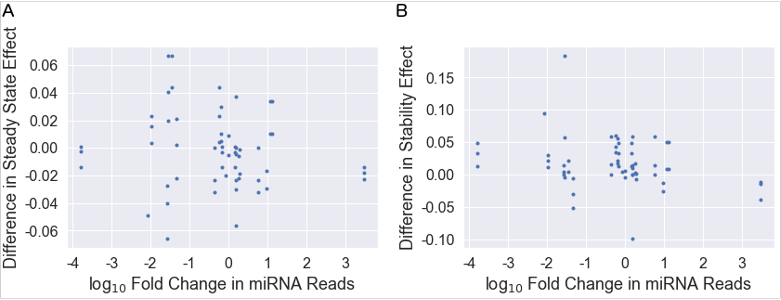

Supplement: jkab404_Supplementary_Figures [file jkab404_supplementary_figures.zip › GENETICS-G3-2021-402854-s19.tif]

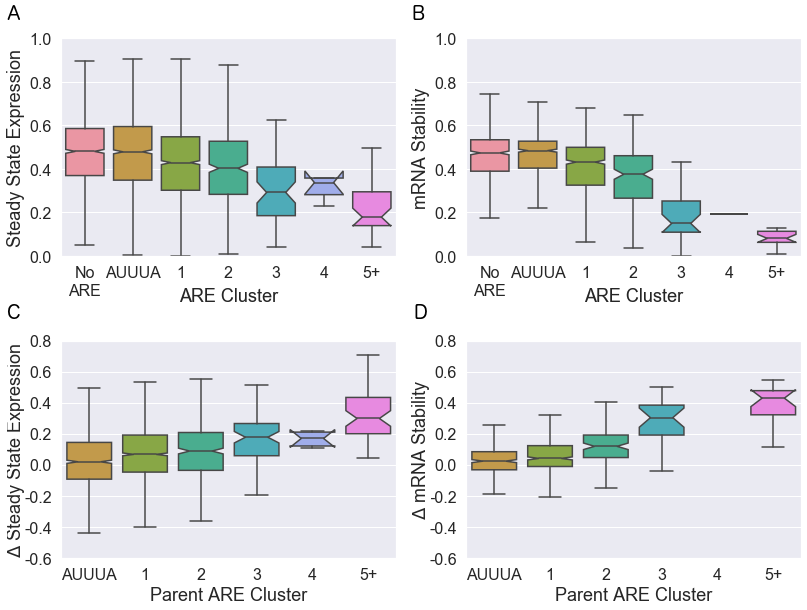

Supplement: jkab404_Supplementary_Figures [file jkab404_supplementary_figures.zip › GENETICS-G3-2021-402854-s20.tif]

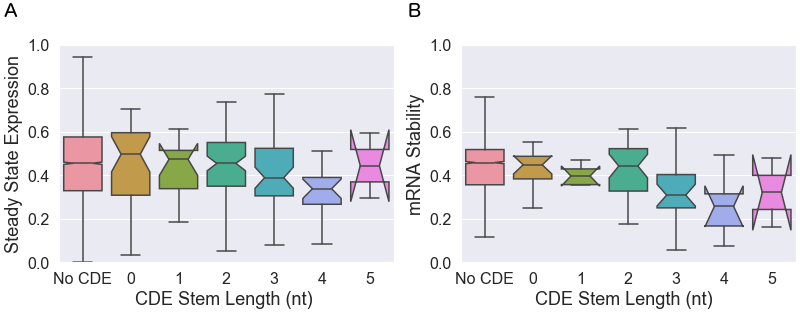

Supplement: jkab404_Supplementary_Figures [file jkab404_supplementary_figures.zip › GENETICS-G3-2021-402854-s21.tif]

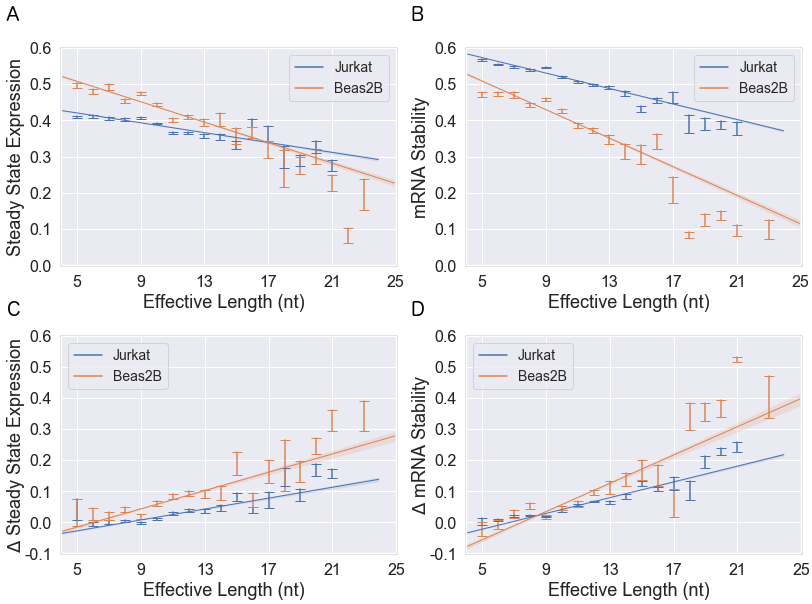

Supplement: jkab404_Supplementary_Figures [file jkab404_supplementary_figures.zip › GENETICS-G3-2021-402854-s22.tif]

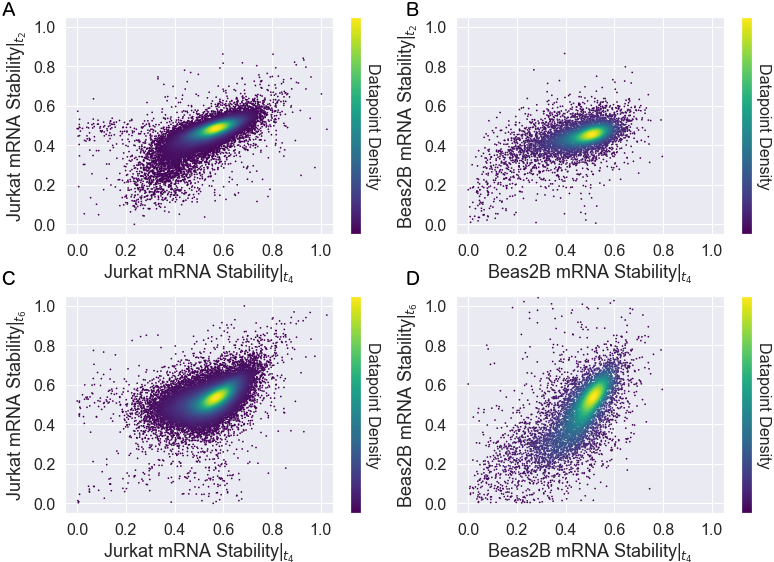

Supplement: jkab404_Supplementary_Figures [file jkab404_supplementary_figures.zip › GENETICS-G3-2021-402854-s23.tif]

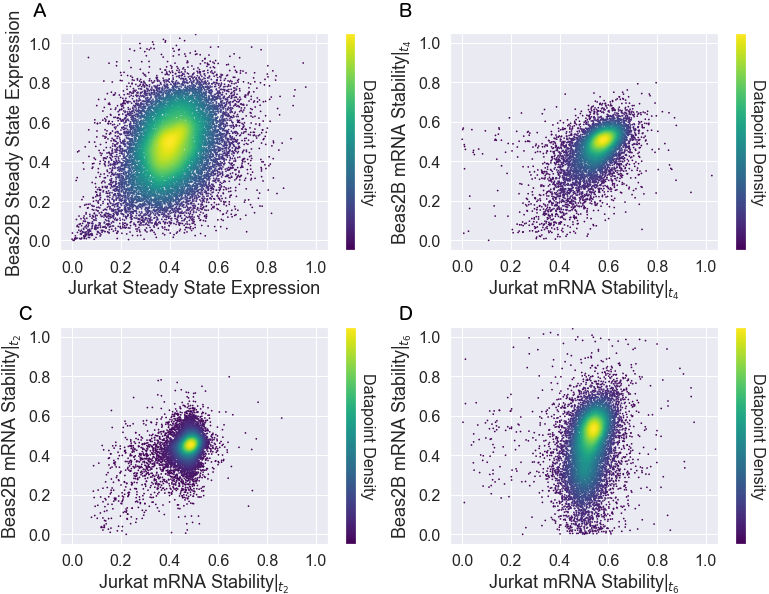

Supplement: jkab404_Supplementary_Figures [file jkab404_supplementary_figures.zip › GENETICS-G3-2021-402854-s24.tif]

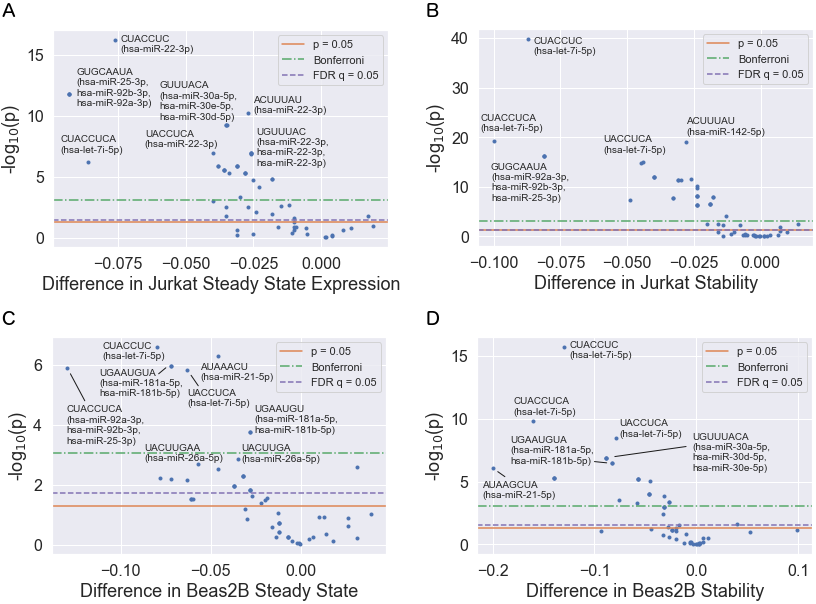

Supplement: jkab404_Supplementary_Figures [file jkab404_supplementary_figures.zip › GENETICS-G3-2021-402854-s25.tif]
