# Supplementary material for: Massively parallel analysis of human 3′ UTRs reveals that AU-rich element length and registration predict mRNA destabilization
Source: G3 (Bethesda). 2021 Nov 29;12(1):jkab404. doi: 10.1093/g3journal/jkab404 (PMC8728028; doi:10.1093/g3journal/jkab404)
Supplement: jkab404_Supplementary_Figures-Tables-Captions [file jkab404_supplementary_figures-tables-captions.pdf]

OXFORD
G3
Genes | Genomes | Genetics

Massively parallel analysis of human 3′ UTRs reveals that AU-rich element length and registration predict mRNA destabilization

David A. Siegel*[,1,†], Olivier Le Tonqueze[1,†], Anne Biton[1,2,†], Noah Zaitlen[1] and David J. Erle[1]

[1]Lung Biology Center, Department of Medicine, University of California San Francisco, 1550 4th St, San Francisco, CA 94158, USA
[2]Hub de Bioinformatique et Biostatistique – Département Biologie Computationnelle, Institut Pasteur, Université de Paris, Bioinformatics and Biostatistics Hub, F-75015 Paris, France
[†]These authors contributed equally to this work.

*David.Siegel@UCSF.edu

Abstract

Keywords:

Supporting Information

S1 File. Supplementary Methods Text containing descriptions of: library preparation and sequencing; plasmids; production of the fast-UTR library; lentivirus production; lentivirus titering; cell culture; clonal isolation of tTa cell lines; transduction and purification; fast-UTR assays; sequencing libraries; read mapping and alignment; barcode misreads; and a description of alternative prediction methods.

S1 Table. ARE Class and Cluster definitions from ARED and ARED-Plus(Bakheet 2001; Bakheet *et al.* 2017). * Indicates 3′ UTR segments containing an AUUUA sequence but not conforming to the class or cluster motifs.

S2 Table. Correlation between predicted and actual gene expression for Jurkat T cell fast-UTR results Calculation was performed for Nth order polynomials using leave-one-out cross validation. We used N=5 for our analysis since higher order polynomials provide diminishing returns.

S3 Table. Lasso amplitudes of the 20 most positive and negative 5-mers for mRNA stability in Jurkat cells. Most positive and negative 5-mers are listed (AUUUA in bold), as well as their Lasso amplitude and number of observations. Directly identified RBP motifs according to the CISBP-RNA database (Ray *et al.* 2013) containing the given 5-mers are listed as well.

S4 Table. Lasso amplitudes of ARE, ARE-like, and other sequences in Jurkat cells.

S5 Table. Out-of-chromosome correlations between predictions and measured data for several ARE categories or simple prediction methods. The columns represent (1) gene expression, (2) stability; (3) the change in steady state expression and the change in stability due to deliberate mutations, respectively. Rows give different ARE categorization or prediction methods. The first three methods are existing methods of ARE categorization in the literature; the next three are naive categorization choices. The next several methods make use of both the ARE length and registration as important parameters. The lasso is trained on the full dataset (it is not ARE-specific) but predictions are tested only on sequences with AREs.

S6 Table. Out-of-chromosome correlations between predictions and measured data for Beas2B. The columns represent (1) gene expression, (2) stability; (3) the change in steady state expression and the change in stability due to deliberate mutations, respectively. Rows give different ARE categorization or prediction methods. The first three methods are existing methods of ARE categorization in the literature; the next three are naive categorization choices. The next several methods make use of both the ARE length and registration as important parameters. The lasso is trained on the full dataset (it is not ARE-specific) but predictions are tested only on sequences with AREs. In the end we show training and testing on different cell lines: "Jurkat ⇒ Beas2B" means the Lasso was trained on Jurkat data and tested on Beas2B; "Beas2B ⇒ Jurkat" is the reverse.

S7 Table. Pearson correlation between prediction method and $t_{1/2}$ for full-length UTRs in Refs (Tani *et al.* 2012; Dölken *et al.* 2010). Columns give different measurements of mRNA lifetimes from different cell lines and references. Rows give different ARE predictions methods for the lifetime of the full-length UTR sequence, focusing on AREScore and Effective Length. Following the methods of AREScore, we report the Pearson correlation between prediction method and $t_{1/2}$ (no leave-one-chromosome out). Negative correlations imply good predictions.

S8 Table. miRNA profiling of Beas2B and Jurkat cells (reads and reads per million) from Reference (Zhao *et al.* 2014). Beas2B results were published in Table S5 of that reference; Jurkat results were unpublished.

S9 Table. Sequence segments that contain some miRNA target sequences have lower stability and steady-state expression miRNAs that made up more than 1% of reads in Beas2B cells in Ref (Zhao *et al.* 2014) were tested for associated target sequences according to the TargetScan database (Agarwal *et al.* 2015). Columns give the miRNA name, the reads per million in Beas2B cells, the target sequences, the number of sequences segments containing

that target sequence (N), as well as the effect size and p-value from regression.

S10 Table. Performance of Some Classification Methods Using Different Time Points Comparison of stability measurements for T2, T4, T6, for three ARE prediction methods for Jurkat cells. Stability predictions for AREs using T2 are better than those using T4, which are better than those using T6.

S11 Table. Performance of a Random Forest Model Variable importance and out of chromosome correlations for a random forest model for steady state expression and stability for Jurkat cells, using R package "ranger" (Wright and Ziegler 2017).

S12 Table. Comparison of previously examined differentially expressed motifs across cell types Effect is the difference between cell types (Jurkat-Beas2B). Steady state and stability measurements use all sequences in the assay (ARE or non-ARE), while delta steady state and stability use only sequences with deliberate ARE mutations.

S13 Table. Effects of miRNAs, ARE Cluster, and CDE Stem Length on Steady State Expression and Stability The mean difference in steady state expression or stability in Jurkat and Beas2B cells between sequences with the given motif and sequences without them in our dataset.

S1 Fig. Steady state expression and stability vary as a function of GC content. Each dot represents one sequence, with a yellow-hot color scale given by the density of surrounding data points calculated with a Gaussian kernel and the highest-density points plotted last. The red line is a moving average of 80 $3'$ UTR segments; the orange line is the 5th-order polynomial fit that we used to residualize the data based on , which shows minimal improvement with higher-order polynomials. Panels (A-B) show Jurkat data and Panels (C-D) show Beas2B.

S2 Fig. mRNA steady state expression as a function of ARE length and registration for Jurkat data. Linear regression gives two-sided p-values of 3×10^{-71}, 6×10^{-56}, 8×10^{-8}, and 0.5, respectively.

S3 Fig. mRNA stability, given as a function of ARE length and registration for Beas2B data. Linear regression gives two-sided p-values of $< 1 \times 10^{-300}$, 9×10^{-268}, 4×10^{-10}, and 0.3, respectively.

S4 Fig. mRNA steady state expression as a function of ARE length and registration for Beas2B data. Linear regression gives two-sided p-values of 8×10^{-71}, 2×10^{-38}, 1×10^{-9}, and 0.4, respectively.

S5 Fig. Distribution of conserved and not conserved elements for Jurkat (A-B) and Beas2B (C-D). Panels (A) and (C) show the change in steady state, while Panels (B) and (D) show the change in stability. Comparing these distributions with a Mann-Whitney U test gives p-values of 0.46, 0.24, 0.21, and 0.27, respectively. Within the group of conserved elements, regressing on LOD score gives p-values of 0.36, 0.74, 0.37, and 0.34, respectively.

S6 Fig. Jurkat data showing mRNA steady state expression and stability, given as a function of ARE length and registration. Panels (A-D) show steady-state expression (A,C) and stability (B,D), respectively, while Panels (E) and (F) show the effect of mutation on steady-state expression and stability, respectively. Values inside of boxes indicate standard errors. The x-axis gives the registration of the start of the ARE in (A,B) and (E,F), and the end of the ARE in (C,D). The y-axis is the length of the ARE, assuming no mismatches. The color scale in (A-D) is determined by setting white to be the mean of all sequences with no ARE, and for (E-F) by setting white to zero.

S7 Fig. Beas2B data showing mRNA steady state expression and stability, given as a function of ARE length and registration. Panels (A-D) show steady-state expression (A,C) and stability (B,D), respectively, while Panels (E) and (F) show the effect of mutation on steady-state expression and stability, respectively. Values inside of boxes indicate standard errors. The x-axis gives the registration of the start of the ARE in (A,B) and (E,F), and the end of the ARE in (C,D). The y-axis is the length of the ARE, assuming no mismatches. The color scale in (A-D) is determined by setting white to be the mean of all sequences with no ARE, and for (E-F) by setting white to zero.

S8 Fig. Distribution of Lasso amplitudes for mRNA stability (Jurkat data). The Lasso amplitude is the predicted magnitude of the effect of a 5-mer on mRNA stability; K-mers with positive/negative amplitudes contribute to a relative increase/decrease in mRNA stability. The peak at 0 is a common feature of Lasso regression.

S9 Fig. Negligible Difference Between CDE Mutation Types. (A) Change in steady state expression vs CDE stem length, separating 2 nt mutation from full shuffle mutation. (B) Change in stability vs CDE stem length. In (C) and (D), for each reference sequence we subtract the steady state expression and stability (respectively) of the two mutant sequences. (C) The distribution of the difference in steady state expression is centered near zero (0.009 ± 0.010). (D) The distribution of differences in stability between CDE mutation types is also centered near zero (-0.011 ± 0.018). Uncertainty is reported as 95% confidence intervals.

S10 Fig. Comparison with Rabani et al. (2017) (Rabani *et al.* 2017). Jurkat data summarizing steady state expression (A,C) and mRNA stability (B,D) for the motifs identified in Rabani et al. Boxplots give medians and quantiles, notches denote confidence intervals. The black horizontal dashed line denotes the grand median over all sequences in this study. Colors denote sequence motifs expected to be "early-onset stabilizing" (EOS), "early-onset destabilizing" (EOD), "late-onset destabilizing" (LOD), "late-onset stabilizing" (LOS), respectively, as defined in the reference. Panels (A,B) are GC-residualized to remove the effects of GC-bias, while panels (C,D) are not. Most of these sequences have very similar effects as the dataset median on average once GC-bias is accounted for, although miR430, ARE, and Pumilio sequences are notable exceptions.

S11 Fig. Comparison with Rabani et al. (2017) (Rabani *et al.* 2017). Beas2B data version of the previous Fig.

S12 Fig. Effect of Padding Sequence AREs The CXCL7 padding sequence has an ARE that was incorporated into the sequence segment for 22 genes. (A) and (B) are copies of Figure S2 A and B, respectively; panels (C) and (D) have removed these AREs from their respective datasets, for comparison.

S13 Fig. Comparison of Different Time Points Jurkat data for mRNA stability and change in stability data constructed from different time points. (A,C,E) show mRNA stability as a function of the length of the ARE and registration; (B,D,F) show the change in mRNA stability as a function of mutation. (A,B) show T2/(T2+T0); (C,D) show T4/(T4+T0); (E,F) show T6/(T6+T0).

S14 Fig. Comparison of Different Time Points, Length Violin Plots Jurkat data for mRNA stability and change in stability data constructed from different time points. (A,C,E) show mRNA stability as a function of the length of the ARE; (B,D,F) show the change in mRNA stability as a function of mutation. (A,B) show T2/(T2+T0); (C,D) show T4/(T4+T0); (E,F) show T6/(T6+T0).

S15 Fig. Position Dependence of AREs Jurkat (A,C,E) and Beas2B (B,D,F) data for mRNA stability and steady state expression as a function of position. In (A-D) the data is broken down by ARED-Plus cluster. Performing linear regression on each cluster data, p-values for clusters 1-5 in (A) were 0.55, 1.6×10^{-5}, 0.35, 0.54, and 0.93, respectively; in (B) they were 0.12, 0.97, 0.43, 0.16, 0.14; in (C) they were 0.00060, 0.041, 0.60, 0.97, 0.15; in (D) they were 0.012, 0.92, 0.47, nan (only two datapoints), and 0.22, respectively. (E) and (F) show data from the two clusters in (A) and (B), respectively, with the smallest p-values. For (E) the slope, pvalue, and rvalue are -0.00052, 1.6e-5, and -0.18, respectively; for (F) the slope, pvalue, and rvalue are -0.00041, 0.00060, -0.099, respectively.

S16 Fig. Effect of miRNA Abundance on Activity Across Cell Types (A) Difference in steady state effect between cell types (Jurkat-Beas2B) from as a function of the differential miRNA read count (log10(miRNA reads per million Jurkat / reads per million Beas2B)). (B) Difference in stability effect between cell types as a function of the differential miRNA read count. Here "steady state effect" and "stability effect" correspond to the effect of a sequence segment containing vs not containing a miRNA target sequence of . If the difference in steady state effect is large, then the proposed target sequence has a greater effect on one cell type than the other. If the differential miRNA read count is large, then that miRNA is also more abundant in one cell type than the other. We expect a negative correlation for destabilizing miRNAs.

S17 Fig. Effect of established ARE clusters on mRNA expression and stability in Beas2B cells This is the Beas2B version of Figure 2. p-values from linear regression are 3.3×10^{-35}, 2.3×10^{-68}, 1.3×10^{-33}, and 7.8×10^{-52}, for panels a-d, respectively.

S18 Fig. Effect of CDE stem length on mRNA expression This is the Beas2B version of Figure 4 A and B. p-values from linear regression (two-sided) are $p = 0.026$ and $p = 0.0015$, respectively. Not enough sequences were present in the Beas2B data to reproduce panels 4C and D.

S19 Fig. Comparison of ARE activity between Jurkat and Beas2B Beas2B cells showed a larger response than Jurkat cells to the presence of AREs in a 3'UTR segment. (A) Steady state expression, (B) stability, (C) change in steady state expression due to deliberate mutations, and (D) change in stability due to deliberate mutations, as a function of the ARE "effective length" (ARE length plus starting registration). Error bars give standard errors. Linear fits are shown to each dataset as a guide to the eye. AREs are more active in Beas2B in each case, with 2-sided p-values of 6×10^{-28}, 5×10^{-36}, 3×10^{-70}, and 7×10^{-17}, respectively (Paternoster *et al.* 1998).

S20 Fig. Comparison of mRNA Stability Between Time Points In this manuscript mRNA stability is defined in Eq. 2, with a ratio of RNA/(RNA+DNA) taken at $t_4/(t_4+t_0)$. In this Figure we show how the mRNA stability compares when defined at different time points, e.g. $\text{mRNA Stability}|_{t_i} \equiv t_i/(t_i+t_0)$. (A) mRNA Stability$|_{t_2}$ vs mRNA Stability$|_{t_4}$ in Jurkat cells, (B) mRNA Stability$|_{t_2}$ vs mRNA Stability$|_{t_4}$ in Beas2B cells, (C) mRNA Stability$|_{t_6}$ vs mRNA Stability$|_{t_4}$ in Jurkat cells, (D) mRNA Stability$|_{t_6}$ vs mRNA Stability$|_{t_4}$ in Beas2B cells. Each datapoint is one distinct sequence. The Pearson correlations between the datasets are 0.68, 0.54, 0.48, 0.66, respectively. All data shown after GC-correction.

S21 Fig. Comparison of Steady State Expression and mRNA Stability Between Cell Lines (A) Steady state expression in Beas2B cells vs Jurkat cells. (B) mRNA Stability$|_{t_4}$ in Beas2B vs Jurkat cells. (C) mRNA Stability$|_{t_2}$ in Beas2B vs Jurkat cells. (D) mRNA Stability$|_{t_6}$ in Beas2B vs Jurkat cells. Each datapoint is one sequence. The Pearson correlations between datasets are 0.32, 0.53, 0.40, 0.22, respectively. All data shown after GC-correction.

S22 Fig. Volcano Plots of miRNA Activity The results in are given as volcano plots, -$\log_{10}$(p) vs difference in (A) Jurkat steady state expression, (B) Jurkat stability, (C) Beas2B steady state, and (D) Beas2B stability. Differences are measured between sequences containing given miRNA target sequence motifs and all other sequences.

Literature cited

Agarwal V, Bell GW, Nam JW, Bartel DP. 2015. Predicting effective microRNA target sites in mammalian mRNAs. eLife. 4.

Bakheet T. 2001. ARED: human AU-rich element-containing mRNA database reveals an unexpectedly diverse functional repertoire of encoded proteins. Nucleic Acids Research. 29:246–254.

Bakheet T, Hitti E, Khabar KSA. 2017. ARED-plus: an updated and expanded database of AU-rich element-containing mRNAs and pre-mRNAs. Nucleic Acids Research. 46:D218–D220.

Dölken L, Malterer G, Erhard F, Kothe S, Friedel CC, Suffert G, Marcinowski L, Motsch N, Barth S, Beitzinger M *et al.* 2010. Systematic analysis of viral and cellular MicroRNA targets in cells latently infected with human -herpesviruses by RISC immunoprecipitation assay. Cell Host & Microbe. 7:324–334.

Paternoster R, Brame R, Mazerolle P, Piquero A. 1998. Using the correct statistical test for the equality of regression coefficients. Criminology. 36:859–866.

Rabani M, Pieper L, Chew GL, Schier AF. 2017. A massively parallel reporter assay of 3′ UTR sequences identifies in vivo rules for mRNA degradation. Molecular Cell. 68:1083–1094.e5.

Ray D, Kazan H, Cook KB, Weirauch MT, Najafabadi HS, Li X, Gueroussov S, Albu M, Zheng H, Yang A *et al.* 2013. A compendium of RNA-binding motifs for decoding gene regulation. 499:172–177.

Tani H, Mizutani R, Salam KA, Tano K, Ijiri K, Wakamatsu A, Isogai T, Suzuki Y, Akimitsu N. 2012. Genome-wide determination of RNA stability reveals hundreds of short-lived noncoding transcripts in mammals. Genome Research. 22:947–956.

Wright MN, Ziegler A. 2017. ranger: A fast implementation of random forests for high dimensional data in c and r. Journal of Statistical Software. 77.

Zhao W, Pollack JL, Blagev DP, Zaitlen N, McManus MT, Erle DJ. 2014. Massively parallel functional annotation of 3′ untranslated regions. Nature Biotechnology. 32:387–391.
