# Supplementary material for: Massively parallel analysis of human 3′ UTRs reveals that AU-rich element length and registration predict mRNA destabilization
Source: G3 (Bethesda). 2021 Nov 29;12(1):jkab404. doi: 10.1093/g3journal/jkab404 (PMC8728028; doi:10.1093/g3journal/jkab404)
Supplement: jkab404_Supplementary_Data [file jkab404_supplementary_data.zip › GENETICS-G3-2021-402854-s03.zip!/G3TemplateSubmission/G3Logo_Vert.pdf]

G3

Genes | Genomes | Genetics
